# Effect of Intrinsic and Extrinsic Motivations on Service Performance after Parental Leave

**DOI:** 10.3390/ijerph19052715

**Published:** 2022-02-25

**Authors:** Yerim Lee, Haeok Liz Kim, Sunghyup Sean Hyun

**Affiliations:** 1 School of Tourism, College of Social Sciences, Hanyang University, 222 Wangsimni-ro, Seongdong-gu, Seoul 04763, Korea; jarnellee@hotmail.com; 2 Computational Social Science Center, Hanyang University, 222 Wangsimni-ro, Seongdong-gu, Seoul 04763, Korea; lette0704@hanyang.ac.kr

**Keywords:** married female flight attendants, intrinsic motivation, extrinsic motivation, job satisfaction, service performance

## Abstract

This study was conducted to empirically investigate the effects of intrinsic and extrinsic motivation on job satisfaction perceived by married female flight attendants after reinstatement, the effect of job satisfaction on service performance after reinstatement, and the moderating effect of the marriage period on the relationship between intrinsic and extrinsic motivation. A questionnaire survey was conducted to collate data from 248 married female flight attendants who had been reinstated after parental leave. The data was analyzed quantitatively, and the main results and implications of this study were as follows. First, intrinsic motivation related to job, aptitude significantly affected job satisfaction, whereas self-realization did not. Second, extrinsic motivation, lay over, salary, and external recognition had significant positive effects on job satisfaction, while welfare did not affect job satisfaction. Third, job satisfaction perceived by married female flight attendants had a significant effect on their service performance after reinstatement. This demonstrates that there is a need to support married women to induce high job satisfaction and for them to reach a level of service performance similar to or better than before they took leave. Fourth, an analysis of the moderating effect of the marriage period on the relationship between intrinsic and extrinsic motivation demonstrated that the marriage period only significantly moderated the relationship between salary and job satisfaction. The study is related to the quality of life and subjective well-being including mental health of service workers in tourism and hospitality. The results of this study can be widely used as reference materials for successful reinstatement, job re-adjustment, job satisfaction, and commitment of all married female employees, especially flight attendants.

## 1. Introduction

According to statistics on parental leave from Statistics Korea, the number of female workers taking parental leave has increased from 87,339 in 2015 to 105,105 in 2019 [1]. With the steady increase in women’s participation in economic activities over the past 20 years, the number of female workers taking parental leave has increased proportionally. According to the Economically Active Population Survey, the participation of women increased by 3.5% over nine years, from 49.6% in 2010 to 53.1% in 2019 [2]. The increase in women’s participation in economic activities over nine years (2010–2019, 3.5 percentage) is much lower than their parental leave over four years (2015–2019, 20.4 percentage), which is interpreted to be due to the continuously supplemented and strengthened parental leave system since the 2000s as society’s demand for better welfare measures for women and children strengthened [3]. Despite the active supplementation of parental leave at the government level, differences have been reported in the parental leave and reinstatement rates by industry and business type [4]. Therefore, many women cannot return to work immediately after parental leave and face difficulties in reemployment because their careers are interrupted involuntarily or voluntarily [5]. According to the “2016 Survey on Economic Activity of Women with Career Interruptions, etc.” by the Ministry of Gender Equality and Family, 46% of married women aged 25–54 years have been interrupted in this way. The main cause of such a high rate of career interruptions has been determined as “life events” experienced by women, such as marriage, childbirth, childrearing, and housekeeping [6].

The airline industry, which traditionally has a high percentage of female employees, has a higher rate of parental leave and successful reinstatement after parental leave of female employees than other industries. This is a noteworthy and unusual phenomenon, considering the controversial recent trend of married women’s lack of reinstatement and subsequent career interruptions. According to an industry report, among the 5640 female flight attendants at Korean Air, 2540 (45.6%) were married, while among the 3157 female flight attendants at Asiana Airlines, 1383 (43.8%) were married. Despite such a high percentage of married female flight attendants, Korean Air and Asiana Airlines encourage the active use of legal maternity protection systems such as parental leave, maternity leave, and family care leave. Consequently, the average usage rates of parental leave at the two airlines were 95% and 95.4%, respectively [7]. This number is overwhelmingly higher than that of other industries, indicating that the airline industry might aid and support married female flight attendants’ parental leave and reinstatement with a flexible and open perspective.

The reason behind this exemplary and encouraging attitude is interpreted as follows. For cabin services, one of the core tasks of airlines, the experience of flight attendants is of great importance because of the nature of the job. Thus, as it is more cost-effective to maintain and manage skilled mid- to long-service flight attendants than hiring and training new flight attendants, preference is naturally given to experienced married female crew members. Because of the nature of the airline industry, where flight attendants’ work skills, flexible service response, and competent service provision are very important, the support and management of competent married female flight attendants with extensive experience and work skills are essential strategies for improving airline business performance and brand awareness [8]. This appears to explain the high rates of parental leave and reinstatement of married female flight attendants. However, many married female flight attendants experience difficulties balancing work and life because of the more irregular work schedules than in other jobs [9,10,11,12]. To supplement and resolve such problems and create and develop an ideal work environment for married female flight attendants to immediately be reinstated after parental leave and an organizational culture of gender equality, the airline industry must develop work and institutional support measures at the airline industry level. The industry must also investigate and analyze the intrinsic and extrinsic motivation affecting the job satisfaction and service performance of female flight attendants reinstated after parental leave and actively reflect these in the workplace. The successful reinstatement and career continuation of experienced and competent married female flight attendants are necessary and desirable for the airline industry and society, but there have been almost no studies on married female flight attendants’ job satisfaction, service performance, and influencing factors after their reinstatement.

This study aims to analyze the intrinsic and extrinsic job motivation of married female flight attendants reinstated after parental leave and their effects on job satisfaction and service performance after reinstatement. First, research was conducted on the relationship of influence on job satisfaction and service performance, focusing on theoretical considerations on intrinsic and extrinsic motivation (Section 2). Based on the previous research, we developed the research hypothesis and research model, as well as a survey questionnaire (Section 3). Through the moderating effect of marriage period, we conducted a statistical analysis of the effect of intrinsic and extrinsic motivation on job satisfaction and service performance (Section 4). This study’s results suggest the necessity of effective theoretical and practical guidelines and reference materials for organizational support and strategies for married female flight attendants’ stable reinstatement and readaptation to the job, workplace, and organization (Section 5). 

Finally, through such analyses based on the results and conclusions of this study, this study presents implications for policy that will help improve the business performance of the airline industry and the utilization and career management of Korean married female employees (Section 6). 

## 2. Theoretical Background

### 2.1. Parental Leave

The parental leave system refers to a system that guarantees and supports married male and female employees in maintaining their employment status without engaging in work for a certain period for childrearing [13]. From the perspective of employees, the parental leave system is a supportive workfare system that guarantees their employment when they must focus only on childrearing for a certain period while also constituting an employment promotion and career support system that guarantees women a stable working life after marriage and childbirth [14]. From the employer’s (company’s) perspective, it is an effective human resource management system that can prevent competent employees from neglecting their job or leaving the company due to childrearing issues [15]. Issues such as ensuring the company’s effective human resource management, maintaining the employment of women of economically active ages, and preventing career interruptions are important social and public issues [16]. Subsequently, it is crucial to seek ways to improve married women’s reinstatement after parental leave and adaptation to the job after reinstatement. In this vein, this study targets the airline industry, which is exemplary in embracing and supporting the reinstatement of married female flight attendants and their adaptation to the job after reinstatement to determine the factors that influence the job satisfaction and service performance of married female flight attendants after reinstatement.

### 2.2. Intrinsic Motivation

The term “motivation,” derived from the Latin verb movere “to move,” is explained as the mental drive that causes a behavior or that determines the direction and characteristic of the behavior [17]. In this sense, understanding motivation is the basis for characterizing individual and organizational behavior [18]. Motivation is divided into intrinsic and extrinsic aspects based on the characteristics or content of various factors that cause a behavior [19]. In Latin, intrinsic motivation is ‘preparationem cordis intrinsecus’. Intrinsic motivation refers to performing certain behaviors for pleasure or satisfaction [20]; the behavior itself leads to an individual’s sense of pleasure, reward, and achievement [21,22]. Intrinsic motivation that boosts job performance refers to the autonomous will, passion, interest, and subjective pleasure internally perceived by various organizational employees [23,24], which is a key factor in promoting a sense of belonging to the organization and job commitment [25]. The more competent the employees, the more pleasure and fulfillment they will feel in their autonomous selection and performance of tasks based on their confidence in their capabilities [21]. In this sense, intrinsic motivation can improve the level of attention and interest in the job [26]. This study aims to analyze the effect of intrinsic motivation of married female flight attendants reinstated after parental leave on their job satisfaction and service performance, referring to a study [27] that presented aptitude and self-realization as sub-factors of intrinsic motivation.

#### 2.2.1. Self-Realization

Goldstein (1939) emphasized the innate potential and inclination toward self-realization [28], which is the foundation of human personality, and defined self-realization as an individual’s behavior that is motivated by one powerful desire rather than multiple desires. Rogers (1995) explained that self-realization is “the tendency to move forward toward maturity, the tendency to express and activate one’s entire abilities, and the tendency to realize their inherent potential” [29] (pp. 25–38). Self-realization serves as a powerful intrinsic motivator for employees of an organization to feel a sense of job satisfaction by committing themselves to their jobs and creating excellent results [30]. Based on the theoretical background, the following hypothesis is derived:

**Hypothesis** **1** **(H1).** *Self-realization has a significantly positive effect on job satisfaction after reinstatement*.

#### 2.2.2. Aptitude

Employees who have selected a career well-matched to their aptitude have been reported to feel a high level of satisfaction with the organization and their job and have lower turnover intentions [31]. Most employees who perform tasks that match their aptitudes have been reported to feel fulfillment from simply being committed to the job and feel a high satisfaction level in working with their co-workers and interpersonal relationships [27]. Lee and Cho (2008) also explained that employees whose work is well suited to their aptitudes have a positive attitude toward their companies due to high job satisfaction [32].

**Hypothesis** **2** **(H2).** *Aptitude has a significantly positive effect on job satisfaction after reinstatement*.

### 2.3. Extrinsic Motivation

In Latin, extrinsic motivation is ‘causam extrinsecam’. Extrinsic motivation is the most representative type of non-autonomous motivation that induces certain behaviors and refers to the behavioral desire to obtain external rewards or results [33]. Individuals who are motivated extrinsically focus on the tangible and material benefits from or rewards for successfully performing certain behaviors that satisfy external demand [22,34]. Extrinsic motivation has been explained as the motive and desire arising from the expectation of tangible and intangible rewards such as promotions, wages, and similar benefits [34]. While intrinsic motivation—attention, interest, reward, and pleasure in the job—leads to subjective satisfaction, extrinsic motivation cannot create satisfaction from the job and thus requires appropriate external and tangible compensation. Additionally, extrinsic motivation can be perceived differently depending on the subjective evaluation and objective standard of the received reward [35]. For airlines, because the financial and material benefits provided for successful job performance are larger than those of other companies [36], extrinsic motivation such as tangible benefits perceived by married female flight attendants are expected to have a significant effect on their job satisfaction and service performance after reinstatement (due to parental leave). In this sense, this study aims to derive valid theoretical and practical guidelines that can help married women be successfully reinstated and readapt to work by analyzing the effects of intrinsic and extrinsic motivation related to the performance of duties by married female flight attendants who have been reinstated after parental leave on job satisfaction and service performance after reinstatement. To this end, this study examined the sub-factors of extrinsic motivation as follows.

#### 2.3.1. Layover

Airline flight attendants must stay in destination countries for a certain period on long-distance overseas flights. Such short-term overseas stays are called layovers, and the necessary costs of stay during a layover are covered by the airline. Due to the nature of the job and company support, airline flight attendants can make good use of their time shopping, traveling, and enjoying their hobbies in overseas countries [37]. A layover is “a pause or stay at a place temporarily” [38] and can be regarded as a special form of layover. Due to the nature of the flight attendant job, repeated short-term stays overseas allow flight attendants to utilize leisure according to their lifestyles efficiently; therefore, repeated overseas experiences due to layovers relieve the stress of their job and enable them to recharge themselves [39]. The personal time granted by layovers is a job benefit that married women in other workplaces do not enjoy. It is also a precious opportunity to free flight attendants from the burden of childcare and allows them to recover their mental freedom, thus serving as an attractive extrinsic motivator that encourages the reinstatement of married female flight attendants and allows them to return smoothly to their jobs after reinstatement.

**Hypothesis** **3** **(H3).** *Layover has a significantly positive effect on job satisfaction after reinstatement*.

#### 2.3.2. Welfare

Welfare refers to non-salary allowances, bonuses, and other non-material support or indirect benefits provided to improve the quality of life for employees and enhance their emotional and social well-being and happiness [40]. The more satisfied employees are with the welfare provided by the company, the higher their organizational commitment, job satisfaction, service orientation, and altruistic behavior toward the organization [41,42]. Various types of welfare are provided for flight attendants, such as priority boarding, support for family occasions, discounts for group companies, childcare fees, school expense support, and workplace daycare. Such diverse welfare benefits greatly affect career or job selection and serve as a key factor in gaining a competitive advantage over other jobs [43]. Another study found that female workers reinstated after parental leave reported that their job satisfaction and emotional state improved when they received quality welfare benefits from their companies [44].

**Hypothesis** **4** **(H4).** *Welfare has a significantly positive effect on job satisfaction after reinstatement*.

#### 2.3.3. Salary

Salaries include monetary and non-monetary compensation paid by companies for employees’ duties, labor, or services [45]. A salary includes basic wages regularly paid to employees, various allowances, and changeable and additional wages such as bonuses, commissions, and incentives paid according to employees’ organizational and departmental performance [46]. Constituent factors of extrinsic motivation in the order of salary, interpersonal relations, flights, and working environment have been demonstrated to impact job satisfaction significantly [47]. This is consistent with another study [48], which reported that salaries have a major effect on the determination of employees’ continuous service. Married employees said that they have a high desire and motivation for continuous service, as continuing to work can help them meet the growing costs of childrearing and living expenses and keep their families economically stable [49]. It has also been reported that economic reasons play a major role in married female employees being reinstated after parental leave [50].

**Hypothesis** **5** **(H5).** *Salary has a significantly positive effect on job satisfaction after reinstatement*.

#### 2.3.4. External Recognition

House (1981) defined external recognition [51] as the interpersonal exchange that includes emotional likeability and support of others, empathy, information about self-assessment, and information about the environment and circumstances. It has been demonstrated that the higher the reputation of or the more aware the employees are of the superiority of the company or organization they work for, the more they tend to identify themselves with the popularity and reputation of the organization. This state of satisfaction from external recognition, such as the social reputation and popularity mentioned above, has an important effect on an individual’s social identity [52,53]. It has been reported that among married female flight attendants, social recognition and recognition and support from their husbands and families have an important effect on their reinstatement after parental leave or smooth readaptation to work [54]. Lee Sungmi (2014) also explained that the psychology of wanting to be recognized by society and family plays an important role in married women deciding to be reinstated [55]. Considering the results of numerous existing studies, this study selected layover, welfare, salary, and external recognition as sub-factors of extrinsic motivation for successful performance of duties or readaptation to work of married female flight attendants reinstated after parental leave.

**Hypothesis** **6** **(H6).** *External recognition has a significantly positive effect on job satisfaction after reinstatement*.

### 2.4. Job Satisfaction and Service Performance

Job satisfaction refers to the overall and subjective satisfaction perceived by the employees in the process of performing their duties [56], which promotes the mutual development of the individual and organization by encouraging a positive attitude toward the work environment and organization [57]. Locke (1976) defined job satisfaction as the overall employee satisfaction with their workplace and job and the pleasure or positive emotional state acquired in the process of performing their duties and performance evaluation [58]. If employees’ job satisfaction is low, their absence and turnover rates increase while their job performance decreases, leading to poor performance and morale throughout the organization [59,60]. Byun (2006) explained that job satisfaction is important because the emotional satisfaction of individual employees is usually reflected in their job and directly linked to productivity [61]. It was reported that the higher the level of job satisfaction perceived by employees, the higher the level of their participation, efforts in their job, and service performance [44]. Herzberg (1996) found that intrinsic motivation, such as pleasure from the work and a sense of achievement and responsibility, have a significant impact on employee job satisfaction [62], which is supported by a study that identified a significant causal relationship between intrinsic motivation and job satisfaction in employees in the travel industry [63]. Intrinsic motivation perceived by employees working in hotels, a service industry, was demonstrated to have a significant effect on job satisfaction [64], while the internal motivation of employees in Korea and China was also found to have a significant effect on job satisfaction [65].

Service performance refers to the “degree to which customer service was successfully performed,” which is one of the core tasks of a company [66] (pp. 103–107). The American Marketing Association defined service as “benefits, actions, and satisfaction provided when selling products,” and thus, the benefit, action, and satisfaction provided to customers determine the employees’ service performance [67]. This illustrates that service performance is a concept that is equated with or closely related to job performance in most companies because customer service is an important core task among the various tasks performed by a company. An organization’s job performance is a key organizational indicator that directly affects organizational commitment and job satisfaction and is a very important concept for both the administrators and employees of the organization [68,69]. As the airline business is centered on cabin services, cabin service performance is a crucial factor in the survival of an airline. In this sense, efficient management of the human resources responsible for cabin services is also an important issue [70]. Hackman and Oldham (1980) found that high job satisfaction and growth satisfaction had a significant effect on the improvement of job performance [71], while Jung and Kim (2007) examined the direct and indirect effects of job satisfaction in a department store as perceived by fashion product sales employees on customer orientation [72]. 

Moreover, a company’s service performance is determined by the response and attitude of employees [73], and the role and attitude of service providers who directly deal with customers are important throughout the entire stages of service production, sales, and consumption [74]. This demonstrates that the job satisfaction of service company employees has an important effect on service performance. In this regard, hotel employees’ job satisfaction has been demonstrated to have an important effect on service performance [75,76]. Various studies have consistently proved that employees’ job satisfaction in the service industry significantly affects job performance, meaning service performance. Thus, this study aims to analyze the effect of job satisfaction on the service performance of married female flight attendants who have been reinstated after parental leave.

**Hypothesis** **7** **(H7).** *Job satisfaction has a significantly positive effect on service performance*.

### 2.5. Marriage Period as Moderation Effect

The conflict married female flight attendants experience at work and home due to their irregular working hours and frequent overseas flights is higher than that experienced by married women in other industries [10]. However, flight attendants who continue to work after marriage or are reinstated after childbirth despite the difficult job and work environment are more likely to be more satisfied with their jobs or greatly fond of and proud of their jobs, or cannot quit their jobs because of financial reasons. This might be related to the high reinstatement rate mentioned earlier. Additionally, the turnover intention was found to be lower in married than unmarried groups, and satisfaction with working conditions, interpersonal relationships, and human resources issues was higher in married groups than in unmarried groups [77]. Married employees will not be able to quit even if they are not satisfied with their jobs or if their loyalty to the organization is low because of their responsibility to support their household. Although Cho and Lee (2015), along with numerous other researchers, have studied the marital status, job satisfaction, and turnover intention of female employees [78], there have been no studies on the job satisfaction and service performance of married female flight attendants according to their marriage period despite the high percentage of working married women and high reinstatement rate in this occupation. Moreover, it has been demonstrated that the shorter the marriage period, the higher the women’s initial job satisfaction, although the rate of increase in their job satisfaction is slower [79]. 

The purpose of this study is to examine the effect of the perceived motivation of reinstated married female flight attendants on their job satisfaction and service performance, and verify whether this effect differs depending on the marriage period, the moderating variable. Based on the marriage period, the following hypotheses were derived:

**Hypothesis** **8a** **(H8a).** *Marriage period play a moderating effect between the self-realization and job satisfaction*.

**Hypothesis** **8b** **(H8b).** *Marriage period play a moderating effect between the aptitude and job satisfaction*.

**Hypothesis** **8c** **(H8c).** *Marriage period play a moderating effect between the layover and job satisfaction*.

**Hypothesis** **8d** **(H8d).** *Marriage period play a moderating effect between the welfare and job satisfaction*.

**Hypothesis** **8e** **(H8e).** *Marriage period play a moderating effect between the salary and job satisfaction*.

**Hypothesis** **8f** **(H8f).** *Marriage period play a moderating effect between the external recognition and job satisfaction*.

## 3. Research Method

### 3.1. Research Hypothesis and Research Model

Existing studies that have analyzed the effect of intrinsic and extrinsic motivation related to the performance of duties on job satisfaction have demonstrated the following. First, Yang and Choi (2011) established the positive effect of intrinsic motivation on job satisfaction among employees of stock companies and explained that the internal sense of achievement and pleasure perceived in the process of performing their duties were key factors in increasing job satisfaction [80]. One study on the relationship between intrinsic motivation and job satisfaction of police officers reported that, of the sub-factors of intrinsic motivation of a sense of achievement from their job and a sense of pleasure from their job, only the sense of achievement from their jobs significantly affected job satisfaction [81], and suggested methods to increase the officers’ sense of achievement. Choi (2020) verified the significant, influential relationship between intrinsic motivation and job satisfaction for hotel employees [26], while Yu and Ju (2018) demonstrated a significant effect of both intrinsic and extrinsic motivation on job satisfaction for health and welfare center employees [35]. Referring to such studies, this study established Hypotheses 1–6, as well as detailed hypotheses about the relationship between intrinsic and extrinsic motivation perceived by married female flight attendants reinstated after parental leave and their job satisfaction as follows. 

Several studies have analyzed the relationship between job satisfaction and service performance. Kim (2019) found that job satisfaction perceived by employees in travel agencies has a significant positive effect on job performance [56], while Cho and Lee (2020) demonstrated a positive effect of job satisfaction on job performance for first-class hotel employees [82]. Ha and Lee (2018) found a significant positive effect of job satisfaction perceived by employees in the food service industry on their job performance [83]. Based on these studies, this study established Hypothesis 7 regarding the relationship between job satisfaction perceived by married female flight attendants reinstated after parental leave and their service performance. 

**Figure 1:**
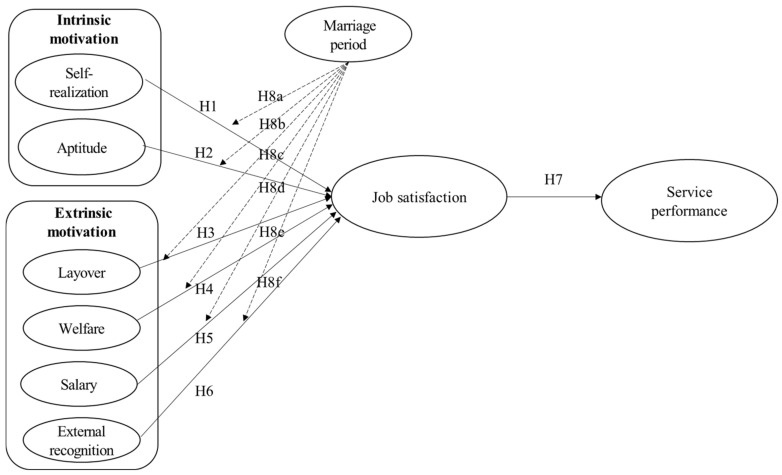
Research model.

Based on the purpose of the study, the relationship between the variables, and the established research hypotheses detailed above, this study established a research model as illustrated in Figure 1.

### 3.2. Questionnaire

To determine the validity of the research hypotheses and model and achieve the research objectives, this study conducted an empirical survey by selecting and extracting detailed measurement elements for independent and dependent variables and each component with reference to numerous studies. The 5-point Likert scale questionnaire survey consisted of five parts: intrinsic motivation, extrinsic motivation, job satisfaction, service performance, and demographic characteristics of married female flight attendants reinstated after parental leave. The details are as follows.

First, tools developed by numerous related prior studies were modified and supplemented in line with this study’s purpose and problem awareness to create two factors and ten items for the measurement of “intrinsic motivation”— six items for “self-realization” [28,29,83] and four items for “aptitude” [31]. Second, tools developed in prior studies were modified and supplemented in line with this study’s purpose and problem awareness to create four factors and 12 items for the measurement of “extrinsic motivation”—three items each for “layover” [83], “welfare” [84], “salary” [45], and “external recognition” [51,52,53,85]. Third, tools developed by Steers (1978) and Price and Mueler (1986) were modified and supplemented in line with this study’s purpose and problem awareness to create five items for the measurement of “job satisfaction” [86]. Fourth, some expressions and phrases of tools developed in other studies [74] were modified and supplemented to create seven items for the measurement of “service performance”.

### 3.3. Data Collection and Analysis Methods

This study selected married female flight attendants of domestic Korean airlines who had been reinstated after parental leave and conducted a survey using a convenience sampling method. The survey was conducted online through social media platforms from 1 February to 10 March 2021. Respondents who received the survey link self-administered the survey. The questionnaire first stated the purpose of the survey and informed the potential respondent that the response will be handled anonymously and confidentially and that he/she may leave the survey at will. No sensitive information beyond demographic characteristics was collected.

At the beginning of the questionnaire, respondents were provided with a clear definition of the term ‘parental leave’ as follow: ‘parental leave’ refers to describe separate family leave available to either parent to care for small children. From the 300 questionnaires distributed, 250 responses were returned (for response rate of 83%). A total of 250 questionnaire surveys were conducted on cabin crew members (excluding interns) who took parental leave. After reviewing the responses, those with missing answer and information were eliminated from the 250 respondents returned. A total of 241 valid questionnaires (excluding nine unreliable responses) were analyzed using the SPSSs Statistics, Amos 21.0 program. 

**Figure 2:**
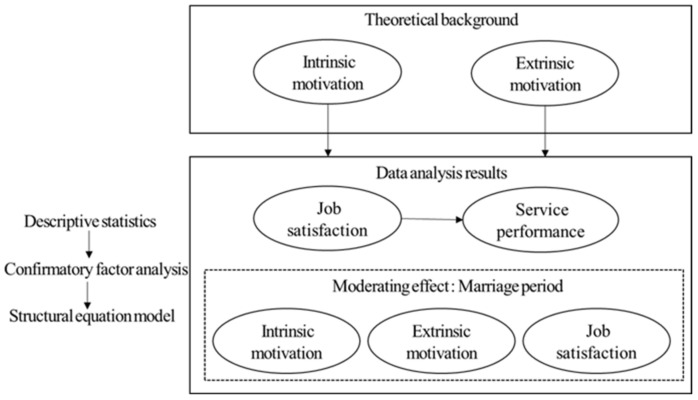
Research methodology.

After coding the collected survey, SPSS was used to sequentially perform a frequency analysis for the demographic characteristics, reliability analysis for each variable (Cronbach’s α), correlation analysis, verification of the structural equation model to verify the hypotheses (Figure 2). Additionally, the study subjects were divided into two groups—those married for nine years or less and those married for more than nine years, to further analyze the differences between the two groups and the moderating effect of the marriage period through multi-group analysis. For multicollinearity, the tolerance limit was 0.512 to 0.627, which was more than 0.1, and the variance expansion index (VIF) was 1.188 to 1.959, not exceeding 10, so correlation between independent variables was not a problem.

## 4. Analysis

### 4.1. Demographic Characteristics of Survey Subjects

The results of the analysis of demographic characteristics of survey subjects are illustrated in Table 1.

### 4.2. Reliability, Validity, and Correlation Analysis of Measurement Variables

First, to verify the reliability of the four variables and each of their sub-factors, their Cronbach’s α was analyzed. In the social sciences, a Cronbach’s α value greater than or equal to 0.6 is considered reliable [87]. According to the reliability analysis results (Table 2), the value of the Cronbach’s α for all variable factors was calculated to be 0.717–0.911, confirming that the variable factors were highly reliable.

Second, Table 3 illustrates the result of the confirmatory factor analysis. To verify the convergent validity of the four variables and each of their sub-factors, the average variance extracted (AVE) and concept reliability (CR) values were analyzed. All factors were above the standard values [88] of 0.5–0.7, verifying their convergent validity. The standard factor capacity for measurement items was significant at the *p* < 0.001 level. The suitability of the model (χ2 = 923.919(df = 461) ***, CFI = 0.906, TLI = 0.892, RMSEA = 0.064) was also verified to be suitable.

Discriminant validity was evaluated by the squared correlations between AVE values and constructs [89]. Third, as illustrated in Table 4, the analysis of the correlation between the variables demonstrates that salary and self-realization (r = 0.192, *p* < 0.05), salary and aptitude (r = 0.129, *p* < 0.05), and salary and layover (r = 0.125, *p* < 0.05) were the only pairs of factors with significant positive correlations. All of the squared correlations between a pair of constructs were lower than AVE. 

### 4.3. Model Suitability and Hypothesis Testing

**Figure 3:**
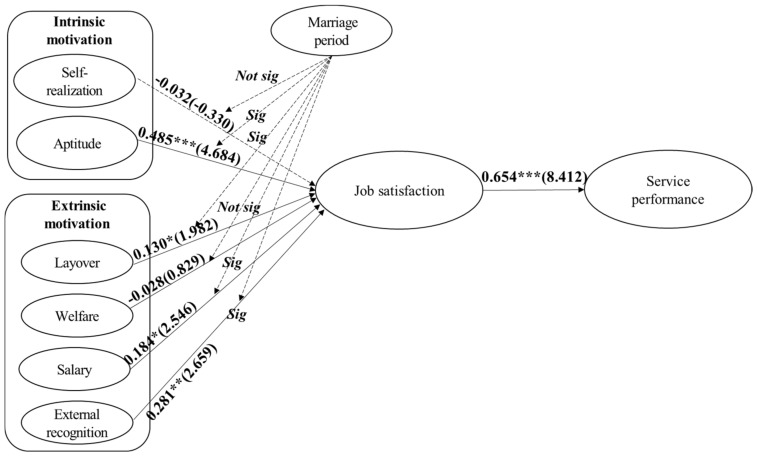
Results of research model (*** *p* < 0.001, ** *p* < 0.01, * *p* < 0.05).

Table 5 and Figure 3 illustrate the results of the hypothesis verification. A path analysis was performed to verify the influential relationships between the four variables and constituent factors. First, the analysis of model suitability (χ2 = 1013.771, df = 472) ***, CFI = 0.890, TLI = 0.877, RMSEA = 0.068) demonstrated that the measurement model for hypothesis testing was suitable. An analysis of the variables-factors relationship demonstrated that among intrinsic motivation, aptitude (β = 0.485, *p* < 0.001) positively affected job satisfaction, while self-realization (β = −0.032, *p* > 0.05) had no effect. Second, among the four sub-factors of extrinsic motivation, external recognition (β = 0.281, *p* < 0.01), salary (β = 0.184, *p* < 0.05), and layover (β = 0.130, *p* < 0.05), in that order, had a positive effect on job satisfaction, while welfare (β = −0.028, *p* > 0.05) was found to have no effect. Third, job satisfaction was found to have a positive effect on service performance (β = 0.654, *p* < 0.001). As a result, the detailed Hypotheses 2, 3, 5, and 6 are supported, meaning that among intrinsic motivation and extrinsic motivation variables is partially supported. 

### 4.4. Analysis of Moderation Effect

This study divided the study subjects into two groups based on their marriage period—more than nine years and nine years or less—and examined the moderating effect of the marriage period on the relationship between intrinsic and extrinsic motivation and job satisfaction (see Table 6). To measure the control variable, the coefficients between the constructs in the AMOS program were estimated, and the value of χ^2^ was calculated. The structural equation model was constrained such that the coefficient between the two groups was the same, the difference between the χ^2^ values was analyzed by comparing the χ^2^ value with the χ^2^ value of each equal constrained model. In the χ^2^ distribution, when the degree of freedom is 1, the level of a = 0.05 is 3.84, which means that if the difference between the χ^2^ values is greater than 3.84, the value of χ^2^ is worse when the two parameters are constrained. Therefore, an unconstrained model is more ideal for analyzing the moderating effect, which indirectly indicates that the difference between the coefficients is significant. According to the moderating effect analysis, the difference between the coefficients of the two groups was statistically significant, as the difference between the χ^2^ values of the constrained and unconstrained models on the effects of extrinsic motivation, salary, and job satisfaction was 4.389. Therefore, it was confirmed that the marriage period significantly moderated the effect of salary, among the extrinsic motivation, on job satisfaction but did not moderate the relationship between other factors and job satisfaction.

A path analysis was performed to verify the influential relationships between the four variables and constituent factors. First, the analysis of model suitability (χ2 = 1013.771(df = 472 ***, CFI = 0.890, TLI = 0.877, RMSEA = 0.068) demonstrated that the measurement model for hypothesis testing was suitable. According to the baseline comparisons, the constrained models were tenable with indices of default model showing good model fit (NFI Delta1 = 0.814, IFI Delta2 = 0.891, TLI rho2 = 0.877, CFI= 0.890). 

The analysis of the variables-factors relationship demonstrated that among intrinsic motivation, aptitude (β = 0.485, *p* < 0.001) positively affected job satisfaction, while self-realization (β = −0.032, *p* > 0.05) had no effect. Second, among the four sub-factors of extrinsic motivation, external recognition (β = 0.281, *p* < 0.01), salary (β = 0.184, *p* < 0.05), and layover (β = 0.130, *p* < 0.05), in that order, had a positive effect on job satisfaction, while welfare (β = −0.028, *p* > 0.05) was found to have no effect. Third, job satisfaction was found to have a positive effect on service performance (β = 0.654, *p* < 0.001). Consequently, Hypotheses H8b, H8c, H8e and H8f are supported, H8a and H8d are not supported.

## 5. Discussion

The purpose of this study was to analyze and confirm the effect of intrinsic and extrinsic motivation for the workplace and job perceived by married female flight attendants who were reinstated after parental leave on their job satisfaction and the effect of job satisfaction on service performance. To this end, this study analyzed the relationships between intrinsic and extrinsic motivation and job satisfaction and between job satisfaction and service performance, and the moderating effect of marriage period on the relationship between intrinsic and extrinsic motivation and job satisfaction for 248 domestic airline flight attendants who are married women. The analysis results and their implications are as follows. 

First, the intrinsic motivation for work perceived by reinstated married female flight attendants was found to have a partially positive effect on job satisfaction after reinstatement. Among the two sub-factors of intrinsic motivation, aptitude (β = 0.485) significantly affects job satisfaction, while self-realization does not have any effect. This means that among the intrinsic and psychological motivation, aptitude has a greater direct significant effect on job satisfaction than self-realization for married female flight attendants reinstated after parental leave. Therefore, to induce smooth readaptation to their jobs and job satisfaction after the reinstatement of married female flight attendants, it is necessary to provide psychological and social encouragement and support at the organizational level so that the reinstated flight attendants can maintain and positively stimulate their curiosity, interest, and aptitude. For instance, new information and knowledge, such as the changing working environment, consumers’ heightened expectations and demands, and the domestic and international movements of the airline industry can be continuously shared and transmitted at the organizational level or through co-workers to flight attendants who are on leave. These updates could help them stay aware of the latest trends in the field and consumer trends, which could induce flight attendants on leave to be aware of their psychological motivation and will for reinstatement. 

Women’s career interruption after marriage and childbirth is not just a problem for individual women but relates to the public problem of using social resources as competent individuals produced through the investment of social and public costs are not being fully utilized [90]. Thus, to minimize the career interruption of married women, a social and public negative and inefficient problem, and the resulting waste of labor, it is necessary to establish a rational and flexible organizational structure and system that can encourage and guarantee married women’s smooth reinstatement and stable readaptation and recommendation to their jobs. To this end, a friendly and rational environment and virtuous cycle must be established in which the reinstated women do not feel a sense of difference, distance, or alienation from their work, workplace, or organization; rather, they feel secure and satisfied after immediately and smoothly being reinstated on-site and adapting to the work, through which they will feel a greater sense of belonging to the organization, solidarity, presence, and loyalty than before taking leave. As the starting point and foundational work to establish such a system and environment, this study empirically verified the significant effect of intrinsic motives perceived by reinstated married women, especially aptitude, on job satisfaction after reinstatement. 

The results above from the analysis of the relationship between intrinsic motivation perceived by reinstated married female flight attendants and job satisfaction are in line with the findings from Pyo (2016), who studied 344 employees working at a hotel, a representative service business, and confirmed that the intrinsic motivation for work has a significant positive effect on job satisfaction [67]; Choi (2020), who verified the positive influential relationship between intrinsic motivation and job satisfaction of hotel employees [26]; Yang and Choi (2011), who confirmed the positive effect intrinsic motivation has on job satisfaction for employees at stock companies [80]; Kim and Kim (2012), who reported a significant effect of intrinsic motivation perceived by team members, who had been coached by the team leader, on job satisfaction for employees in 12 companies [91]; and Yoon and Song (2014), who reported the positive effect of intrinsic motivation on the sense of achievement and pleasure in work in police officers [81]. It can be seen through these results that autonomous and intrinsic interest and curiosity, pleasure, and a sense of achievement perceived by employees in most industries regarding their workplace or jobs result in the positive outcome of job satisfaction. Consequently, it has been repeatedly confirmed that strategies to improve and enhance workplace and job satisfaction, commitment, and organizational support need to be explored by further encouraging employees’ intrinsic motivation. 

Second, the extrinsic motivation related to the workplace, job, and organization perceived by reinstated married female flight attendants was demonstrated to have a partial positive effect on job satisfaction. Among the four sub-factors of extrinsic motivation, external recognition (β = 0.281), salary (β = 0.184), and layover (β = 0.130), in that order, have been found to have a significantly positive effect on job satisfaction, while welfare did not have any effect, indicating that married female flight attendants who had relatively high extrinsic motivation for their workplace and jobs due to various external factors, such as layover, salary, and external recognition, before reinstatement can be satisfied with their job again through smooth reinstatement after taking a leave of absence. It is evident that various external and environmental factors and motivation, including financial and material benefits, must be sufficiently guaranteed to help married women be reinstated stably after a leave of absence and readapt to work. External merits such as a sufficient salary, high social reputation of the job, and support for overseas work positively affect female flight attendants’ job satisfaction after reinstatement, and they are expected to have a desirable effect on the organization’s management of human resources. This may have implications for building a foundation for minimizing and preventing career interruptions in married women.

The above analysis results regarding the relationship between extrinsic motivation perceived by reinstated married female flight attendants and job satisfaction indirectly and partially supports the findings of Pyo (2016), who demonstrated a significant positive effect of extrinsic motivation for the workplace and work on job satisfaction for hotel employees [67]; Yu and Ju (2018), who reported a significant effect of extrinsic motivation for work on job satisfaction for employees of basic mental health welfare centers [35]; and Kim (2019), who verified the positive effect of extrinsic motivation for public service on job satisfaction in public officials at fire departments in Gyeonggi Province [92]. Based on these studies, many industries have been confirmed to induce and boost job satisfaction of their employees, knowing that extrinsic and environmental motivation and benefits related to the workplace and work perceived by their employees support or are mutually organic and complementary with the intrinsic motivation, such as interest, curiosity, pleasure, and a sense of accomplishment in their workplace and jobs. In this sense, it is necessary to establish organization-wide strategies that can improve and promote employees’ workplace, job, organizational satisfaction, commitment, and sense of belonging by further strengthening and encouraging external and material benefits related to their job or work, such as salary, overseas dispatch, and social recognition and preference.

Third, it has been demonstrated that job satisfaction perceived by reinstated married female flight attendants has a significant effect on their service performance after reinstatement (β = 0.654). This means that for married women to continue to perform service tasks after reinstatement in a stable manner similar to or better than before they went on leave, it is necessary to induce and support their job satisfaction after reinstatement. Furthermore, when employees in various industries perceive job satisfaction through various internal, external, and environmental factors, it positively affects the operation performance and customer service of their organization, company, or institution. This result is in line with earlier studies that demonstrated a significant effect of job satisfaction on job performance according to favorable working conditions for travel agency employees [56], a positive effect of job satisfaction on service performance in first-class hotel employees [82], and that job satisfaction positively affects the job performance of employees in the foodservice industry [83]. These studies indicate that the job satisfaction perceived by most employees and workers enhances various forms of job performance, service performance, and operational performance, thereby contributing to the organization, company, and institution’s mid- to long-term development. Keeping this in mind, it is necessary to seek strategies from various angles to promote and induce employees’ job satisfaction and commitment, which will help various organizations, companies, and institutions with their performance enhancement and development in the future.

Fourth, analyzing the mediating effect of married female flight attendants’ marriage period (nine years or less or more than nine years) on the relationship between intrinsic and extrinsic motivation and job satisfaction demonstrated that the marriage period only had a significant mediating effect on the relationship between the extrinsic motivation sub-factor, salary, and job satisfaction. More specifically, the relationship between salary and job satisfaction for female flight attendants who were married for nine years or less was more positive than for female flight attendants who were married for more than nine years. The longer the marriage period, the lower the job satisfaction perceived from the salary. This has been interpreted to mean that, for married women whose marriage period is long and their children are growing up, their job satisfaction from salary is much lower due to their increasing education, child support, and living expenses. Consequently, organizational encouragement and support strategies must be established to encourage the job satisfaction of women married for a long time and prevent stress related to childrearing or other life issues or exhaustion from jobs. 

## 6. Conclusions

From various perspectives, this study analyzed and verified: (1) the effects of intrinsic and extrinsic motivation on job satisfaction for married female flight attendants who were reinstated after parental leave; (2) the effects of job satisfaction after reinstatement on service performance; and (3) the moderating effect of the different marriage periods on the influential relationship between intrinsic and extrinsic motivation and job satisfaction. Subsequently, it established valid theoretical and practical guidelines and reference materials that help derive and establish organizational encouragement and support strategies needed for the stable reinstatement and readaptation to the workplace and jobs of married female flight attendants. Based on the results and conclusions of this study, the following implications can be presented.

First, as an academic implication, although many studies have empirically analyzed the effect of intrinsic and extrinsic motivation perceived by employees of various organizations, companies, and institutions on their job satisfaction and commitment, and research has been abundantly produced in fields such as organizational management, business administration, and public institution policy management, there have been almost no studies that have analyzed the relationship between the variables mentioned above for employees of the airline industry, especially married female flight attendants who were reinstated after parental leave. Through an empirical analysis of this unique subgroup, this study has expanded the scope of research on the relationship between intrinsic and extrinsic motivation for the workplace and work and job satisfaction and segmented and characterized specific analysis topics.

Second, as a practical implication, by analyzing the relationship between intrinsic and extrinsic motivation for work perceived by married female flight attendants and job satisfaction and service performance from various angles, as well as the mediating effect of different marriage periods, this study proposes organizational support and encouragement strategies to help married female flight attendants maintain an active and enthusiastic organizational life after reinstatement to the extent they did before taking leave and the direction for creating a friendly environment. By doing so, this study provides realistic and practical guidelines that can help married women smoothly and stably continue their working life, career, and self-development, minimizing their stress and exhaustion in the airline industry and various other industries and businesses. The results of this study can be used as a valid reference for preventing and eradicating women’s career interruptions due to marriage or childbirth and the resulting loss of society’s workforce.

Despite the theoretical and practical implication, this study has the following limitations. First, this study did not target all reinstated married female flight attendants in the industry but rather adopted a sample survey method that surveyed and analyzed married female flight attendants who voluntarily participated. Thus, the results, conclusions, and implications of this study must be generalized with caution. Second, the intrinsic, extrinsic, and environmental variables that affect job readaptation, job satisfaction, and commitment of married female flight attendants or married female employees may be more diverse, but this study did not consider the various variables or differences between individuals. Professional measurement tools should be developed to demonstrate in-depth, detailed characteristics, situations, and individual differences related to reinstatement and readaptation of married female employees. Lastly, the data was collected 248 in South Korea. When conducting analysis of structural equation modeling, a sample size 200 is considered acceptable for reliable conclusion and making accurate statically [93]. However, since the survey conducted in South Korea, the extent to which the results are generalizable may be limited cross-culturally.

In future research, a broader sample survey should be conducted than that conducted in this study, and specialized, in-depth measurement items must be developed that can demonstrate in more detail, from various perspectives, and in a multifaceted manner, the process of reinstatement and readaptation to work by married female employees. Through this, more meaningful research results and conclusions can be drawn.

## Figures and Tables

**Table 1:** Demographic Characteristics of Survey Subjects.

Item		*N* (%)	Item		*N* (%)
Education level	Associates degree	27 (10.9)	Age	20–30	5 (2.0)
	Bachelor’s degree	189 (76.2)		31–35	60 (24.2)
	Master’s degree or higher	32 (12.9)		36–40	65 (26.2)
Position	Flight attendants	68 (27.4)		41–45	74 (29.8)
	Assistant purser	69 (27.8)		More than 46	44 (17.7)
	Over purser	111(44.8)	Income (year)	Less than $4000	45 (18.1)
Working period	Under 5 years	14 (5.6)		Less than $5000	57 (23.0)
	Less than 10 years	54 (21.8)		Less than $6000	65 (26.2)
	Less than 15 years	71(28.6)		More than $6000	81 (32.7)
	More than 15 years	109 (44.0)		Total	248 (100)

**Table 2:** Results of reliability verification.

Variables		Sub-Factors	Cronbach’s α
Independent variables	Intrinsic motivation	Self-realization (6)	0.907
		Aptitude (4)	0.813
	Extrinsic motivation	Layover (3)	0.875
		Welfare (3)	0.775
		Salary (3)	0.717
		External recognition (3)	0.733
Dependent variables		Job satisfaction (5)	0.911
		Service performance (7)	0.867

**Table 3:** Confirmatory factor analysis results.

Variables			Estimate		S.E.	C.R.	*p*	AVE	CR	Cronbach’s α
			B	β						
IM	SR (6)	1	0.797	1				0.673	0.924	0.907
		2	0.86	1.089	0.07	15.525	***			
		3	0.896	1.089	0.066	16.421	***			
		4	0.82	1.053	0.072	14.541	***			
		5	0.652	0.847	0.077	10.937	***			
		6	0.705	0.876	0.073	12	***			
	AT (4)	1	0.809	1				0.596	0.853	0.813
		2	0.801	1.089	0.083	13.111	***			
		3	0.722	0.938	0.08	11.657	***			
		4	0.588	0.819	0.089	9.196	***			
EM	LO (3)	1	0.814	1				0.750	0.900	0.875
		2	0.857	1.061	0.073	14.526	***			
		3	0.833	1.012	0.071	14.149	***			
	WF (3)	1	0.737	1				0.630	0.833	0.775
		2	0.89	1.292	0.11	11.782	***			
		3	0.638	1.068	0.113	9.471	***			
	SL (3)	1	0.669	1				0.501	0.750	0.717
		2	0.736	1.095	0.145	7.57	***			
		3	0.605	0.962	0.133	7.235	***			
	ER (2)	1	0.883	1				0.656	0.789	0.733
		2	0.663	0.876	0.093	9.442	***			
JS (5)		1	0.707	1				0.671	0.911	0.843
		2	0.717	1.132	0.11	10.298	***			
		3	0.725	0.998	0.096	10.407	***			
		4	0.767	1.043	0.095	10.952	***			
		5	0.702	1.088	0.108	10.09	***			
SP		1	0.811	1				0.775	0.959	0.912
		2	0.874	1.086	0.067	16.287	***			
		3	0.876	1.142	0.07	16.345	***			
		4	0.793	1.062	0.075	14.127	***			
		5	0.854	1.082	0.069	15.721	***			
		6	0.693	0.977	0.082	11.875	***			
		7	0.518	0.803	0.096	8.361	***			

Note: *** *p* < 0.001, IM: intrinsic motivation, SE: self-realization, AT: aptitude, LO: layover, WF: welfare, SL: salary, ER: external recognition, JS: job satisfaction, SP: service performance.

**Table 4:** Results of correlation analysis between variables.

	SR	AT	LO	WF	SL	ER	JS	SV
SR	1							
AT	0.613 (0.376)	1						
LO	0.420 (0.176)	0.443 (0.196)	1					
WF	0.437 (0.191)	0.474 (0.225)	0.316 (0.100)	1				
SL	0.192 (0.037)	0.129 (0.017)	0.125 * (0.016)	0.347 (0.120)	1			
ER	0.542 (0.294)	0.433 (0.187)	0.286 (0.082)	0.492 (0.242)	0.320 (0.102)	1		
JS	0.494 (0.244)	0.589 (0.347)	0.392 (0.154)	0.429 (0.184)	0.280 (0.078)	0.519 (0.269)	1	
SP	0.463 (0.214)	0.456 (0.208)	0.356 (0.127)	0.373 (0.139)	0.238 (0.057)	0.466 (0.217)	0.595 (0.354)	1
AVE	0.673	0.596	0.75	0.63	0.501	0.656	0.671	0.775

Note: IM: intrinsic motivation, SE: self-realization, AT: aptitude, LO: layover, WF: welfare, SL: salary, ER: external recognition, JS: job satisfaction, SP: service performance. * *p* < 0.05.

**Table 5:** Results of hypothesis verification.

Path			β	B	S.E.	C.R.	*p*	Hypothesis
SR	→	JS	−0.032	−0.022	0.067	−0.33	0.741	Not Supported
AT	→	JS	0.485	0.365	0.078	4.684	0.000 ***	Supported
LO	→	JS	0.13	0.091	0.046	1.982	0.048 *	Supported
WF	→	JS	−0.028	−0.024	0.069	−0.346	0.729	Not Supported
SL	→	JS	0.184	0.16	0.063	2.546	0.011 *	Supported
ER	→	JS	0.281	0.2	0.075	2.659	0.008 **	Supported
JS	→	SP	0.654	0.622	0.074	8.412	0.000 ***	Supported

Note: *** *p* < 0.001, ** *p* < 0.01, * *p* < 0.05, IM: intrinsic motivation, SE: self-realization, AT: aptitude, LO: layover, WF: welfare, SL: salary, ER: external recognition, JS: job satisfaction, SP: service performance.

**Table 6:** Results of the moderation effect analysis.

Variable	Constrained Model χ2 (df)		Free Model χ2 1 (df)	χ2 2 − χ2 1 (Critical Value: 3.84)	Estimate	
					9 Years or Less	More than 9 Years
SR	JS	1760.619(954)	1760.619(944)	0.000	−0.098	−0.089
AT		1760.638(954)		0.019	0.52	0.476
LO		1764.433(954)		3.814	0.011	0.247
WF		1760.868(954)		0.249	−0.052	0.039
SL		1765.008(954)		4.389	0.234	−0.068
JS		1760.664(954)		0.045	0.36	0.381

Note: IM: intrinsic motivation, SE: self-realization, AT: aptitude, LO: layover, WF: welfare, SL: salary, ER: external recognition, JS: job satisfaction.
